# Beyond Social Exchange: Career Adaptability Linking Work Stressors and Counterproductive Work Behavior

**DOI:** 10.3389/fpsyg.2019.01079

**Published:** 2019-05-14

**Authors:** Kun Yu, Chang Liu, Yuhui Li

**Affiliations:** School of Labor and Human Resources, Renmin University of China, Beijing, China

**Keywords:** career adaptability, work stressors, counterproductive work behavior, perceived organizational support, career construction theory, job demand-control-support model

## Abstract

Drawing upon career construction theory (Savickas, 2002, 2013) and the job demand-control-support model (JDCS; Johnson and Hall, 1988; Van der Doef and Maes, 1999), the present study aims to explore the adaptability resources mechanism of the relationship between work stressors and counterproductive work behavior (CWB). Two-wave data were collected from 305 employees working in the operation department of an e-commerce company. The results showed that career adaptability mediated the relationship between work stressors and CWB against both coworkers (CWB-I) and the organization (CWB-O), going above, and beyond the mediation effect of job satisfaction (i.e., an indicator of a social exchange path). Also, the association between career adaptability and CWB-O was stronger among employees who perceived a low (vs. high) level of organizational support. This study sheds light on how work stressors are related to CWBs indirectly through career adaptability. The findings also offer practical advice for organizations to prevent CWBs by developing employees’ adaptability.

## Introduction

Work stress is one of the oldest and most endurable topics in organizational research field (Rodell and Judge, 2009) and has drawn considerable attention in recent years (Scheck et al., 1997; Wallace et al., 2009), mainly because of its detrimental effect to both employees, and the organization (Cooper and Marshall, 1975; Hall and Savery, 1987; Webster et al., 2010). As a set of obstacles in employees’ work environment, such as role conflict, role ambiguity (Kahn et al., 1964), and situational constraints (Peters and O’Connor, 1980), work stressors cause employees’ illness (Jex et al., 1992), anxiety, depression, and exhaustion (Jex, 1998). Work stressors also reduce positive outcomes at work, leading to a low level of job satisfaction (Beehr et al., 2001), job commitment, and task performance (Cooper et al., 2001).

Besides, many researchers are interested in the relationship between work stressors and the negative forms of work behavior (Fox et al., 2001; Spector and Fox, 2005; Fida et al., 2015), such as counterproductive work behavior (CWB; Bennett and Robinson, 2000). CWB is a voluntary behavior that goes against organizational norms and harms the organization or its members (Robinson and Bennett, 1995; Bennett and Robinson, 2000; Spector et al., 2006). Social exchange theory (Blau, 1964) that emphasize the reciprocity of social-emotional benefits is one of the prevailing theories adopted to explain the relationship between work stressors and CWB (Penney and Spector, 2005; Spector and Fox, 2005; Harold et al., 2016). Under the social exchange framework, CWB can be seen as a response representing unpleasant exchange to the work stressors that interfere with employees’ job goals (Penney and Spector, 2005; Spector and Fox, 2005; Harold et al., 2016).

Conceptually, CWB consists of two sub-dimensions on the basis of the target, namely CWB against coworkers (CWB-I) and CWB against the organization (CWB-O; Robinson and Bennett, 1995). The target similarity model of social exchange (Lavelle et al., 2007) posits that individuals would “return the favor” to the same foci that the favor (or harm) comes from. It may be reasonable to argue that CWB-O is a retaliative response to the organization for employees who are experiencing work stressors because the organization is the source of work stressors (such as situational constraints; Peters and O’Connor, 1980). On the contrary, from the social exchange framework, CWB against their coworkers (i.e., CWB-I) may not happen when encountering work stressors because coworkers are not the source of work stressors. However, the negative relationship between work stressors and CWB-I was found in several studies (e.g., Fox et al., 2001; Fida et al., 2015). The mismatch between the social exchange explanation and empirical findings calls for a better theoretical framework to link work stressors and the two forms of CWB.

The first purpose of the present research is to address the above theoretical void and link work stressors and CWB from the career construction perspective (Savickas, 2002, 2013). Career construction theory denotes that individuals’ development over their career is not driven by the maturation of inner structures but by adaptation to an environment (Savickas, 2013). According to career construction theory, career adaptability, which refers to an individual’s psychosocial resources to cope with the ill-defined problems, and unfavorable working conditions (Savickas, 1997), is a core concept and the linking mechanism underlying the relationship between personal and/or situational factors and adaptation outcomes (e.g., performances). Based on career construction theory (Savickas, 2002, 2013), we first argue that work stressors can drain individuals’ psychosocial resources for dealing with unfavorable working conditions (i.e., career adaptability; Savickas, 1997). Moreover, CWB, whether coworkers- or organization-targeted, can be a result of a lack of adaptability resources, such as lacking self-regulatory resources to control inappropriate actions against coworkers or the organization (Savickas, 2002, 2013). In short, we argue an adaptability resources mechanism to better understand the relationship between work stressors and both forms of CWB. Specifically, we propose career adaptability as a mediator to link work stressors and CWB-O, going above and beyond the mediating effect of job satisfaction, an indicator of social exchange (Bateman and Organ, 1983). Also, we argue that career adaptability also plays a mediating role in the relationship between work stressors and CWB-I, in which job satisfaction may no longer play a mediating role.

**FIGURE 1:**
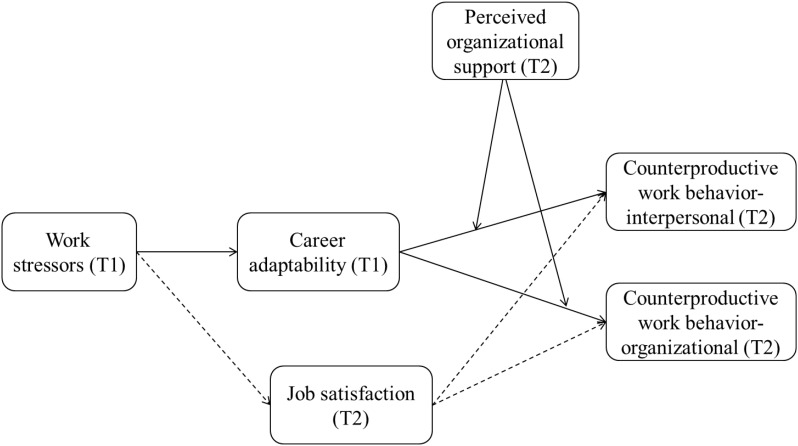
Hypothesized research model. The solid lines between work stressors and CWBs indicate the hypothesized adaptability paths, while the dotted lines indicate the controlled social exchange paths.

The second purpose of the current study is to explore possible boundary conditions in the indirect relationship between work stressors and CWB via career adaptability. Based on the job demand-control-support model (JDCS; Johnson and Hall, 1988; Van der Doef and Maes, 1999), we conceptualize employees’ career adaptability as resources for self-regulation and argue that it will compensate for the insufficient supply of resources from outside on inhibiting strain response (e.g., CWBs), such as perceived organizational support (POS), which refers to an employees’ perception that their organization values their contribution, and cares about their well-being (Eisenberger et al., 1986). Specifically, we propose that a low level of POS will strengthen the negative relationship between career adaptability and both CWB-I and CWB-O. To sum up, we aim to test a moderated mediation model in the current study, see Figure 1.

### Work Stressors and Career Adaptability

Based on career construction theory (Savickas, 2002, 2013), career adaptability indicates “an individual’s resources for coping with current and anticipated tasks, transitions, traumas in their occupational roles” (Savickas and Porfeli, 2012, p. 662). Career adaptability is psychosocial strengths or capacities that individuals can use to cope with the complex or unfamiliar situations and solve the problems presented in the working settings (Savickas and Porfeli, 2012). Specifically, career adaptability consists of four adaptive strategies: concern, control, curiosity, and confidence. Concern is the orientation to and involvement in preparing for the future. Control is the self-discipline, effort, and persistence shown in making decisions. Curiosity is the openness to explore circumstances and seek for opportunities. Lastly, confidence is the efficacy to solve problems and overcome obstacles (Savickas, 2002).

As a psychosocial construct, career adaptability is more unstable than the personality traits (Savickas, 2013) and could be developed through learning (Brown, 2015) and training (Koen et al., 2012) and predicted by factors from both inner and outer worlds (Savickas and Porfeli, 2012). Indeed, researchers have shown that individual factors, including emotional intelligence (Coetzee and Harry, 2014), sense of control (Duffy, 2010), future work self (Guan et al., 2014), proactivity, core self-evaluations (Hirschi et al., 2015), hope, and optimism (Wilkins et al., 2014) appear to predict career adaptability resources. Some positive contextual factors were also found to be predictors of career adaptability resources, which includes career-specific parental behaviors (Guan et al., 2015), positive relationships with parents (Soresi et al., 2014), and social support (Tian and Fan, 2014; Wang and Ying, 2015).

As career adaptability is the psychosocial resources to cope with job demands, especially complex or unfavorable problems and obstacles (Savickas, 2013), we propose that work stressors, which include a set of unfavorable working conditions such as role conflict, role ambiguity (Kahn et al., 1964), and situational constraints (Peters and O’Connor, 1980), could reduce employees’ career adaptability by draining their psychosocial resources. First, under high levels of work stressors, employees will experience uncertainty about their work (Lazarus, 1991). It may be harder for employees to make clear job plans (i.e., a high level of concern). Second, employees are likely to perceive a high level of work stressors (e.g., role ambiguity) as unmanageable and beyond their control (Wallace et al., 2009). In other word, work stressors may be a detriment to employees’ control over their actions (i.e., a high level of control). Third, under a high level of work stressors, employees will perceive that their opportunity for personal growth is threatened. As a result, they will invest less time and energy on the exploration of surroundings (i.e., a high level of curiosity). Finally, under a high level of work stressors, individuals will appraise the working environment as a barrier to achieve job goals (Lazarus, 1993), and experience negative emotions such as frustration (Berkowitz, 2003). Thus, it may harm employees’ self-efficacy on job performance (i.e., a high level of confidence). To sum up, we argue that work stressors would be negatively associated with career adaptability. Thus, we predict:

Hypothesis 1: Work stressors are negatively associated with career adaptability.

### The Mediating Role of Career Adaptability Linking Work Stressors and Counterproductive Work Behavior

Based on career construction theory (Savickas, 2002, 2013), we argue that career adaptability is negatively related to both CWB-I and CWB-O for three reasons. First, individuals with a high level of career adaptability are more future-oriented (Savickas, 2002), which means that they are less likely to get frustrated by immediate obstacles in the work settings. At the same time, the experience of frustration is a critical cause of CWB (Harold et al., 2016). Therefore, individuals with a high level of career adaptability will show fewer frustration responses and have a low level of CWB, whether against coworkers or the organization. Second, individuals with a high level of career adaptability will have more control over their actions (Savickas, 2002), and are more capable of suppressing possible impulsive or inappropriate behaviors. Thus, with a high level of career adaptability, individuals are less likely to adopt CWB, whether against coworkers or the organization. Thirdly, successful regulation of the self contributes to many adaptive outcomes in society, such as success at school, at college, and in the workplace (Hagger et al., 2010). As an important self-regulatory resource in work settings (Savickas, 2013), career adaptability may help individuals show a good self in the workplace by preventing them from performing CWB.

According to career construction theory, there is a chain of putative effects: Adaptivity → Adaptability → Adapting → Adaptation (Savickas, 2005). Career adaptability, as psychosocial resources, is the linking mechanism underlying the relationship between adaptivity (e.g., dispositional constructs) and adaptation outcomes (e.g., performances). Career adaptability could develop through both inner and outer worlds (Savickas and Porfeli, 2012), which means that both contextual factors, and personal factors may affect individuals’ career adaptability (Savickas, 2013). Therefore, it is reasonable to argue that career adaptability could also explain the relationship between situational factors and work outcomes from a career construction perspective. Indeed, researchers have found that career adaptability mediates the relationship between social support and job satisfaction (e.g., Han and Rojewski, 2015), and between job demands and employee engagement (Tladinyane and Van der Merwe, 2016).

Therefore, combining the above reasoning, we expect that career adaptability will, at least partially, mediates the relationship between work stressors, and CWB. Specifically, we propose that individuals under a high level of work stress would drain their psychosocial resources, which in turn would lead to a higher level of CWBs. In summary, we propose that career adaptability plays a mediating role in the relationship between work stressors and CWB-I and the CWB-O:

Hypothesis 2: Career adaptability mediates the positive relationship between work stressors and (a) counterproductive work behavior-interpersonal and (b) counterproductive work behavior-organizational.

Moreover, from the social exchange perspective (Blau, 1964), for the unfavorable outcomes that a high level of work stressors leading to, employees may be dissatisfied with the organization (which is the source of the work stressors), and “return the favor” by adopting CWB-O, including behaviors such as enjoying excessive breaks or withholding work effort (Bennett and Robinson, 2000). In this case, job satisfaction may mediate the relationship between work stressors and CWB-O, based on the norm of reciprocity in social exchanges (Blau, 1964). The mediating effects of job satisfaction and career adaptability may act as a dual process to explain the relationship between work stressors and CWB-O. In other words, we propose that career adaptability will mediate the work stressors-CWB-O relationship above and beyond the mediation of job satisfaction.

However, while coworkers are not the cause of role conflict, role ambiguity or situational constraints, there should not be a social exchange relationship between work stressors and CWB-I, including behaviors such as making fun of or withholding crucial information from coworkers (Bennett and Robinson, 2000). To put in another way, we do not expect job satisfaction to mediate the relationship between work stressors and CWB-I. The mediating effect of career adaptability may act as the only valid path to explain the relationship between work stressors and CWB-I in the current study. This also means that career adaptability mediates the work stressors-CWB-I relationship above and beyond the mediation of job satisfaction. Taken together, we predict:

Hypothesis 3a: Career adaptability mediates the positive relationship between work stressors and counterproductive work behavior-interpersonal above and beyond the mediation of job satisfaction.
Hypothesis 3b: Career adaptability mediates the positive relationship between work stressors and counterproductive work behavior-organizational above and beyond the mediation of job satisfaction.

### The Moderating Role of Perceived Organizational Support

Perceived organizational support (POS), based on the Job demand-control-support model (Johnson and Hall, 1988), is an essential resource that individuals can use to buffer against job strain (House et al., 1988; Thoits, 1995). Thus, under a high level of POS, individuals would have additional resources to help them cope with the strain. For that CWB is a typical behavioral strain response (Fox et al., 2001), POS might, therefore, help individuals cope with the strain and perform less CWB. Therefore, POS may substitute for the effect of career adaptability on CWB.

Specifically, when individuals perceive a high level of organizational support, they will receive enough attention and caring from the organization and can easily get help from the organization when needed (Johnson and Hall, 1988). The support from the organization may provide enough resources for individuals to cope efficiently with their job stress and maintain control over their behavior, preventing them from acting with hostility toward coworker, or the organization. In this case, individuals may no longer need their own adaptability resources to achieve control over behaviors. In other words, although career adaptability is expected to affect CWB, the negative career adaptability-CWB relationship may be weaker when POS is at a high level.

In contrast, under a low level of POS, individuals will receive less attention and caring from the organization, and cannot get needed help from the organization (Johnson and Hall, 1988). This means that the organization provides less additional resources to help individuals cope with a stressful situation. Under such circumstance, individuals are less likely to depend on resources from outside. Instead, they will be more likely to rely on their own adaptability resources to cope with the stressful situation. In other words, the negative relationship between career adaptability and CWB will be stronger when POS is low. Thus, we predict:

Hypothesis 4a: Perceived organizational support moderates the relationship between career adaptability and counterproductive work behavior-interpersonal, such that the negative relationship is stronger when perceived organizational support is low rather than high.
Hypothesis 4b: Perceived organizational support moderates the relationship between career adaptability and counterproductive work behavior-organizational, such that the negative relationship is stronger when perceived organizational support is low rather than high.

As we proposed that career adaptability mediates the work stressors–CWB relationship after incorporating job satisfaction as a competing mediator, we expect that POS, as resources from outside, will moderate the indirect effect of work stressors on both CWB-I and CWB-O via career adaptability. This leads to the following moderated mediation hypotheses:

Hypothesis 5a: Perceived organizational support moderates the mediated relationship between work stressor and counterproductive work behavior-interpersonal via career adaptability, such that the mediated relationship is stronger when perceived organizational support is low rather than high.
Hypothesis 5b: Perceived organizational support moderates the mediated relationship between work stressor and counterproductive work behavior-organizational via career adaptability, such that the mediated relationship is stronger when perceived organizational support is low rather than high.

## Materials and Methods

### Participants and Procedure

Data for the present study were obtained from employees working in the operation department of an e-commerce company in China. Employees in the operation department were in charge of customer service and product promotion. Five hundred employees agreed to participate, and each received a printed survey package. Every survey package includes an instruction letter and a Time 1 questionnaire. At Time 1, participants’ work stressors and career adaptability were measured. One month later, Time 2 questionnaires were distributed to those participants, and we used an identification code to link every participant’s two-wave surveys. At Time 2, participants’ job satisfaction, POS, and CWB were measured. Among them, 305 employees completed surveys in both two waves (response rate = 61%). Among the 305 participants, 46.9% were men. At Time 1, the average age of the participants was 28.3 years (*SD* = 3.9).

### Measures

We used a translation-back translation procedure (Brislin, 1980) to translate all items measured from English to Chinese. Participants were asked to indicate the extent to which they agreed with each item based on their experiences at work on a 7-point Likert scale (from 1 = “*Strongly disagree*” to 7 = “*Strongly agree*”) unless otherwise noted.

#### Work Stressors

Work stressors were rated by employees at Time 1 using the 5-item hindrance dimension of Cavanaugh et al. (2000) job stressor measure. Participants were asked to rate how much stress following items were causing them using a Likert scale (from 1 = “produces no stress” to 5 = “produces a great deal of stress”). A sample item is “The inability to clearly understand what is expected of me on the job.” Higher scores represent a higher level of work stressors. The Cronbach’s α of the scale for the current data set was 0.78.

#### Career Adaptability

We measured career adaptability at Time 1 using the 24-item Career Adapt-Abilities Scale (CAAS) developed by Savickas and Porfeli (2012). The CAAS includes four dimensions, namely concern, control, curiosity, and confidence. Sample items for the above four dimension are “Thinking about what my future will be like,” “Taking responsibility for my actions,” “Looking for opportunities to grow as a person,” and “Working up to my ability,” respectively. Higher scores represent a higher level of career adaptability. The Cronbach’s α of each of the four dimensions was 0.78 for concern, 0.82 for control, 77 for curiosity, and 0.83 for confidence. Cronbach’s α of the overall CAAS scale was 0.94.

#### Perceived Organizational Support

Perceived organizational support was measured at Time 2 by the six-item POS scale (Eisenberger et al., 2001). A sample item is “The organization really cares about my well-being.” Higher scores represent a higher level of POS. The Cronbach’s α of the scale for the current data set was 0.89.

#### Counterproductive Work Behavior

Employees’ CWB was measured at Time 2 using a 19-item scale developed by Bennett and Robinson (2000). The scale contains two dimensions, namely CWB against the coworkers (CWB-I; 7 items) and CWB against the organization (CWB-O; 12 items). We used Bennett and Robinson (2000) measure of workplace deviance instead of Spector et al. (2006) CWB scale because we focus mainly on the two dimensions of deviant behaviors and the core of these two measures is the same. A sample item of CWB-I is “Said something hurtful to someone at work,” and a sample item of CWB-O is “Taken an additional or longer break than is acceptable at your workplace.” Higher scores represent a higher level of counterproductive behavior. The Cronbach’s α of CWB-I and CWB-O for the current data set was 0.92 and 0.94, respectively.

#### Control Variables

Employees’ gender (1 = male, 2 = female) and age (in years) were controlled in the analysis because they often associate with extra-role work behaviors (e.g., Berry et al., 2007). Besides, employees’ job satisfaction was incorporated as a competing mediator. Job satisfaction was argued to mediate the relationship between extra-role behaviors and their antecedents from a social exchange perspective (e.g., Bateman and Organ, 1983). Job satisfaction was measured by the 6-item job satisfaction scale (Tsui et al., 2011). A sample item is “I am satisfied with the nature of the work I perform.” Higher scores represent a higher level of job satisfaction. Cronbach’s α for job satisfaction in this study was 0.92.

## Results

The means, standard deviations, reliabilities, and correlations among the study variables are presented in Table 1.

**Table 1:** Means, standard deviations, reliabilities, and correlations.

Variable	*M*	*SD*	1	2	3	4	5	6	7	8	9	10	11	12
1. Gender	1.53	0.50	–											
2. Age	28.27	3.90	0.01	–										
3. Work stressors	2.50	0.86	0.07	0.01	(*0.78*)									
4. Career adaptability	4.97	0.98	−0.03	0.03	−0.43^∗∗^	(*0.94*)								
5. CA: concern	4.85	1.05	−0.04	0.04	−0.37^∗∗^	0.88^∗∗^	(*0.78*)							
6. CA: control	5.08	1.11	−0.00	0.03	−0.38^∗∗^	0.92^∗∗^	0.73^∗∗^	(*0.82*)						
7. CA: curiosity	4.92	1.03	−0.04	0.00	−0.40^∗∗^	0.92^∗∗^	0.74^∗∗^	0.78^∗∗^	(*0.77*)					
8. CA: confidence	5.03	1.11	−0.02	0.03	−0.42^∗∗^	0.93^∗∗^	0.73^∗∗^	0.82^∗∗^	0.84^∗∗^	(*0.83*)				
9. Job satisfaction	4.63	1.34	−0.18^∗∗^	0.03	−0.54^∗∗^	0.47^∗∗^	0.46^∗∗^	0.40^∗∗^	0.44^∗∗^	0.40^∗∗^	(*0.92*)			
10. POS	4.38	1.23	−0.15^∗∗^	0.07	−0.48^∗∗^	0.43^∗∗^	0.43^∗∗^	0.36^∗∗^	0.43^∗∗^	0.37^∗∗^	0.84^∗∗^	(*0.89*)		
11. CWB-I	2.42	1.17	−0.04	0.00	0.23^∗∗^	−0.24^∗∗^	−0.15^∗^	−0.29^∗∗^	−0.17^∗∗^	−0.27^∗∗^	−0.23^∗∗^	−0.24^∗∗^	(*0.92*)	
12. CWB-O	2.30	1.10	0.02	−0.01	0.25^∗∗^	−0.34^∗∗^	−0.21^∗∗^	−0.38^∗∗^	−0.29^∗∗^	−0.37^∗∗^	−0.32^∗∗^	−0.31^∗∗^	0.75^∗∗^	(*0.94*)

*N = 305. Reliabilities of scales are in the diagonals where applicable. CA, career adaptability; POS, perceived organizational support; CWB-I, counterproductive work behavior-interpersonal; CWB-O, counterproductive work behavior-organizational; M and SD are used to represent mean and standard deviation, respectively. ^∗^p < 0.05, ^∗∗^p < 0.01.*

### Tests of Measurement Model

A confirmatory factor analysis (CFA) using Mplus 7 (Muthén and Muthén, 2012) was conducted to examine whether the constructs measured in the present study were distinguishable from each other. Specifically, the measurement model included all the measures used in the present study (i.e., six latent variables including Time 1 work stressors, Time 1 career adaptability, Time 2 POS, Time 2 job satisfaction, Time 2 CWB-I, and Time 2 CWB-O). Due to a large number of scale items (i.e., 60 items), the sample size to parameter ratio may be too small and, thus, adversely impacts the standard errors, and stability of the estimates (see Landis et al., 2000). To resolve the problem, we created parcels for study variables that contains subdimensions. Specifically, we created four parcels for career adaptability based on its dimension differentiation.

Confirmatory factor analysis results indicated that the 6-factor measurement model (i.e., all variables are independent of each other) fits the data well, χ^2^(725) = 1407.05, *p* < 0.01, comparative fit index (CFI) = 0.92, Tucker-Lewis Index (TLI) = 0.92, root mean square error of approximation (RMSEA) = 0.06. In fact, the 6-factor model fits the data better than 2-factor model, in which constructs measured in each wave were combined, respectively (i.e., work stressors, career adaptability at Time 1, and job satisfaction, POS, CWB-I, and CWB-O at Time 2), χ^2^(739) = 4363.76, *p* < 0.01, CFI = 0.60, TLI = 0.58, RMSEA = 0.13. The 6-factor model also fits the data better than one-factor model (i.e., all variables were combined), χ^2^(740) = 5323.51, *p* < 0.01, CFI = 0.49, TLI = 0.46, and RMSEA = 0.14. The CFA results provided support for the discriminant validity of the constructs in the present study.

### Tests of Hypotheses

We first tested the mediation model using Mplus 7. First, Time 1 Career adaptability and Time 2 job satisfaction were predicted by Time 1 work stressors. Second, Time 2 CWB-I and Time 2 CWB-O were both predicted by Time 1 Career adaptability and Time 2 job satisfaction. Employees’ gender and age were controlled. The effects of work stressors on CWB-I and CWB-O were also controlled. This model accounted for 19, 31, 11, and 14% of the variance in career adaptability, job satisfaction, CWB-I, and CWB-O, respectively.

**FIGURE 2:**
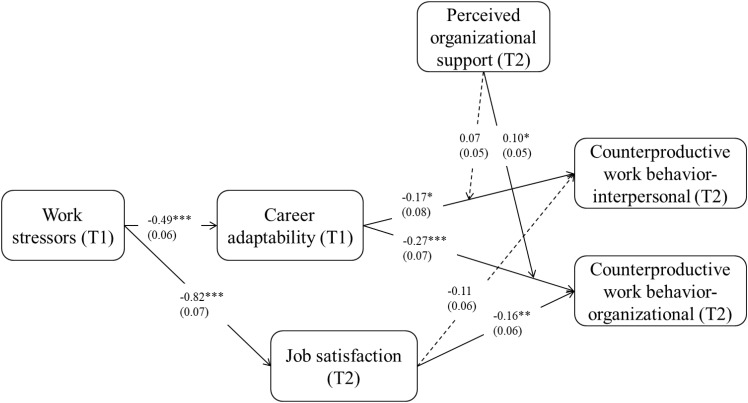
The final model with unstandardized coefficients. ^∗^*p* < 0.05, ^∗∗^*p* < 0.01, ^∗∗∗^*p* < 0.001.

As shown in Table 2 and Figure 2, the effect of work stressors on career adaptability (*B* = -0.51, *p* < 0.001) was significantly negative. Thus, Hypothesis 1 was supported. The effect of work stressors on job satisfaction (*B* = -0.82, *p* < 0.001) was also significantly negative. In addition, the effect of career adaptability on CWB-I (*B* = -0.17, *p* < 0.05) and on CWB-O (*B* = -0.27, *p* < 0.001) were both significantly negative. These results demonstrated that career adaptability was negatively related to both CWB-I and CWB-O. In contrast, the effect of job satisfaction on CWB-I (*B* = -0.11, *p* > 0.05) was not significant, while the effect of job satisfaction on CWB-O (*B* = -0.16, *p* < 0.01) was significantly negative.

**Table 2:** Regression results.

	Model 1				Model 2			
Predictor	Career adaptability	Job satisfaction	CWB-I	CWB-O	Career adaptability	Job satisfaction	CWB-I	CWB-O
Gender	0.00	−0.38^∗∗^	−0.17	−0.05	0.00	−0.38^∗∗^	−0.16	−0.04
Age	0.01	0.01	0.00	0.00	0.01	0.01	0.00	0.00
Work stressors	−0.49^∗∗∗^	−0.82^∗∗∗^	0.15	0.06	−0.49^∗∗∗^	−0.82^∗∗∗^	0.12	0.02
Career adaptability			−0.17^∗^	−0.27^∗∗∗^			−0.13	−0.21^∗∗^
Job satisfaction			−0.11	−0.16^∗∗^			−0.03	−0.10
POS							−0.10	−0.07
CA^∗^POS							0.07	0.10^∗^
*R*^2^	0.19^∗∗∗^	0.31^∗∗∗^	0.108^∗∗^	0.14^∗∗∗^	0.19^∗∗∗^	0.31^∗∗∗^	0.08^∗∗^	0.13^∗∗∗^

*N = 305. CA, career adaptability; POS, perceived organizational support; CWB-I, counterproductive work behavior-interpersonal; CWB-O, counterproductive work behavior-organizational; SE, standard error. ^∗^p < 0.05, ^∗∗^p < 0.01, ^∗∗∗^p < 0.001.*

We then tested the mediation effect of career adaptability in the relationship between work stressors and both CWB-I and CWB-O. The indirect effect of work stressors on CWB-I via career adaptability was significant [*B* = 0.10, *p* < 0.01, 95% confidence interval (CI = 0.02, 0.17)]. Thus, Hypothesis 2a was supported. Moreover, the indirect effect of work stressors on CWB-O via career adaptability was significant (*B* = 0.16, *p* < 0.001, 95% CI = 0.08, 0.23). Thus, Hypothesis 2b was supported. The above results indicate that career adaptability mediated the relationship between work stressors and both CWB-I and CWB-O. We also test the mediating effect of the four dimensions of career adaptability in the relationship between work stressors and CWBs as a supplementary analysis. The results showed that, first, control (*B* = 0.12, *p* < 0.01, 95% CI = 0.05, 0.21) and confidence (*B* = 0.12, *p* < 0.05, 95% CI = 0.04, 0.22) significantly mediated the stressors-CWB-I relationship, while concern (*B* = 0.04, *p* > 0.05, 95% CI = -0.03, 0.10) and curiosity (*B* = 0.05, *p* > 0.05, 95% CI = -0.02, 0.14) did not. In contrast, in the stressors-CWB-O relationship, all four dimensions, namely concern (*B* = 0.06, *p* < 0.05, 95% CI = 0.003, 0.13), control (*B* = 0.16, *p* < 0.001, 95% CI = 0.08, 0.25), curiosity (*B* = 0.12, *p* < 0.01, 95% CI = 0.04, 0.20) and confidence (*B* = 0.18, *p* < 0.001, 95% CI = 0.09, 0.28) had significant mediation effects.

We then tested the mediation effect of career adaptability in the relationship between work stressors and both CWB-I and CWB-O while incorporating job satisfaction as a competing mediator. The indirect effect of work stressors on CWB-I via career adaptability was significant (*B* = 0.08, *p* < 0.05, 95% CI = 0.01, 0.16). In contrast, the indirect effect of work stressors on CWB-I through job satisfaction was not significant (*B* = 0.09, *p* > 0.05, 95% CI = -0.01, 0.19). Thus, Hypothesis 3a was supported, indicating that career adaptability mediated the relationship between work stressors and CWB-I while job satisfaction did not mediate the relationship. Moreover, the indirect effect of work stressors on CWB-O via career adaptability was significant (*B* = 0.13, *p* < 0.001, 95% CI = 0.06, 0.20). The indirect effect of work stressors on CWB-I through job satisfaction was also significant (*B* = 0.13, *p* < 0.01, 95% CI = 0.04, 0.22). Thus, Hypothesis 3b was supported, indicating that career adaptability mediated the relationship between work stressors and CWB-O while incorporating job satisfaction as a competing mediator. The above results showed that career adaptability mediated the relationship between work stressors and both forms of CWB above and beyond the mediating effect of job satisfaction. Especially, job satisfaction did not mediate the stressors-CWB-I relationship, which was consistent with our hypothesis.

We also did a supplementary analysis testing the mediating effect of the four dimensions of career adaptability in the relationship between work stressors and CWBs after incorporating job satisfaction as a competing mediator. The results showed that, first, control (*B* = 0.07, *p* < 0.05, 95% CI = 0.01, 0.14) and confidence (*B* = 0.07, *p* < 0.05, 95% CI = 0.01, 0.15) have a significantly mediated the stressors-CWB-I relationship, while concern (*B* = 0.01, *p* > 0.05, 95% CI = -0.03, 0.04) and curiosity (*B* = 0.02, *p* > 0.05, 95% CI = -0.03, 0.07) did not. In contrast, in the stressors-CWB-O relationship, control (*B* = 0.08, *p* < 0.05, 95% CI = 0.03, 0.16), curiosity (*B* = 0.05, *p* < 0.01, 95% CI = 0.01, 0.11), and confidence (*B* = 0.10, *p* < 0.01, 95% CI = 0.04, 0.19) had significant mediation effects, while concern (*B* = 0.01, *p* > 0.05, 95% CI = −0.02, 0.06) did not.

We then used a moderation model (Model 2) estimated based on Model 1 to test Hypothesis 4a,b. Specifically, Model 2 included the interaction effect between Time 1 career adaptability and Time 2 POS on Time 2 CWB-I and Time 2 CWB-O. This model accounted for 19, 31, 8, and 13% of the variance in career adaptability, job satisfaction, CWB-I, and CWB-O. The unstandardized coefficient estimates of Model 2 are presented in Table 2.

**FIGURE 3:**
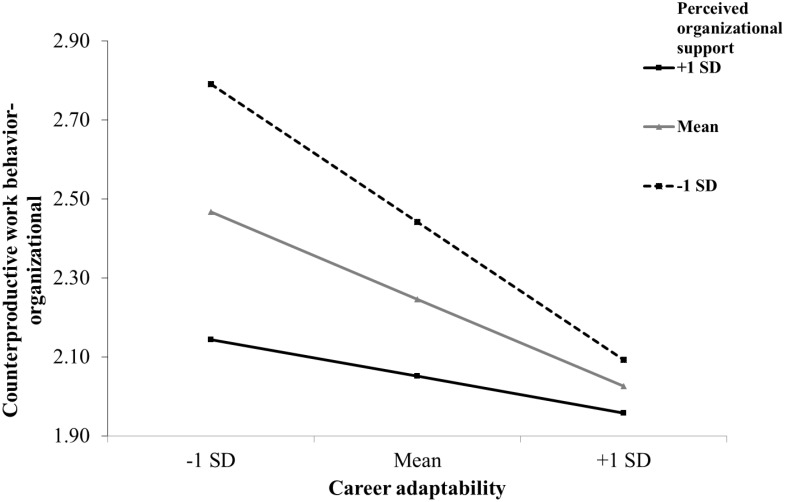
The interaction effect between career adaptability and perceived organizational support on counterproductive work behavior-organizational (CWB-O).

As shown in Table 2 and Figure 2, the interaction effect between career adaptability and POS on CWB-I was not significant (estimate = 0.07, *p* > 0.05). Thus, Hypothesis 4a was not supported. In contrast, the interaction effect between career adaptability and POS on CWB-O was significantly positive (estimate = 0.10, *p* < 0.05). Simple slope tests showed that the relationship between career adaptability and CWB-O was significantly negative for employees under low levels (simple slope = −0.30, *p* < 0.01) and average levels (simple slope = −0.21, *p* < 0.01) of POS but not significant for employees under high levels (simple slope = −0.11, *p* > 0.05) of POS, as shown in Figure 3. Thus, Hypothesis 4b was supported.

Furthermore, moderated mediation indexes firstly showed that POS did not moderate the mediated relationship between work stressors and CWB-I via career adaptability (*B* = −0.06, *p* > 0.05). Specifically, the mediation relationship was significantly positive for employees under low levels (simple slope = 0.09, *p* < 0.05) of POS but not significant for employees under average levels (simple slope = 0.06, *p* > 0.05) or high levels of POS (simple slope = 0.03, *p* > 0.05). Thus, Hypothesis 5a was not supported.

Moreover, POS moderated the mediated relationship between work stressors and CWB-O via career adaptability (*B* = −0.09, *p* < 0.05). Specifically, the mediation relationship was significantly positive employees under low levels (simple slope = 0.15, *p* < 0.01) and under average levels (simple slope = 0.10, *p* < 0.01) of POS but not significant for employees’ high levels of POS (simple slope = 0.05, *p* > 0.05). Thus, Hypothesis 5b was supported.

## Discussion

In the present study, we drew upon career construction theory (Savickas, 2002, 2013) and the job demand-control-support model (JDCS; Johnson and Hall, 1988; Van der Doef and Maes, 1999) and investigated the adaptability resources mechanism of the relationship between work stressors and CWB against both coworkers and the organization. Specifically, time-lagged data were collected from 305 employees to examine whether career adaptability mediates the work stressors-CWB relationship above and beyond the social exchange mechanism indicated by job satisfaction. Moreover, we also examined how POS moderates the indirect relationship between work stressors and both CWB-I and CWB-O via career adaptability.

The findings of the current research supported our mediation hypotheses. Consistent with career construction theory (Savickas, 2002, 2013), we found that career adaptability mediated the relationship between work stressors and both CWB-I and CWB-O, after incorporating job satisfaction as a competing mediator. These results that both career adaptability and job satisfaction mediated the stressors-CWB-O relationship suggest that, in addition to considering exchange response that individuals may have against work stressors (Penney and Spector, 2005; Fida et al., 2015), individuals’ adaptability resources to cope with the environment deserve more research attention. Individuals would have CWB not only because they are not satisfied with the unfavorable treatment (e.g., the work stressors), but also because their psychosocial resource is damaged by the work stressors. Moreover, the results that career adaptability but not job satisfaction mediated the stressors-CWB-I relationship especially supported our argument around CWB against coworkers, as CWB-I is hard to be explained by the social exchange because coworkers are not the sources of stressor, and targets for revenge. It makes more sense to argue that individuals have their adaptability resource depleted because of work stressors and, therefore, cannot confidently exerting control over possible inappropriate behaviors, whether against the organization, or coworkers. The results of the supplementary analysis that control and confidence have significant mediation effects in both stressors-CWB-I and stressors-CWB-O relationships –whether incorporating job satisfaction or not – provided additional evidence on this argument.

Consistent with the JDCS model (Johnson and Hall, 1988; Van der Doef and Maes, 1999), our results also provided support for the moderating role of POS in the relationship between career adaptability and CWB-O. First, we found that POS had a buffering effect on the career adaptability-CWB-O relationship. Furthermore, the results of moderated mediation analysis showed that the indirect effect of work stressors on CWB-O was stronger when employees perceived a low level of organizational support. In contrast, POS moderated neither the relationship between career adaptability and CWB-I nor the indirect relationship between work stressors and CWB-I via career adaptability. These results may be explained by the target similarity model (Lavelle et al., 2007), which emphasizes the target specific characteristics of a social exchange relationship. For instance, POS, as resources from the organization to help individuals, would lead to employees’ organizational citizenship behavior toward the same foci (i.e., the organization) rather than other foci (e.g., coworkers; Lavelle et al., 2007). Similarly, POS might only help individuals exert control over CWB against the same foci (i.e., the organization), buffering the relationship between career adaptability, and CWB-O. On the contrary, POS may not help individuals exert control over CWB against the individual foci, such as coworkers (CWB-I). Thus, POS may not play a moderating role in the relationship between career adaptability and CWB-I.

### Theoretical Contributions

The present research contributes to the literature in three ways. First, we advance the literature on the work stress by introducing an adaptability resources mechanism to the stressor-strain relationship. Previous research has posited that the relationship between work stressors and CWB could be explained under the social exchange framework or its extensions (Penney and Spector, 2005; Fida et al., 2015). We offer an alternative way of understanding how work stressors trigger an individual’s anti-social behavior toward coworkers and the organization. The adaptability resources mechanism is better in explaining the stressors-CWB-I relationship than the social exchange mechanism. By incorporating indicator of social exchange (i.e., job satisfaction) in the research model, the current study is the first to reveal how career adaptability mediated the work stressors-CWB relationship above and beyond the social exchange mechanisms.

Second, based on the career construction theory (Savickas, 2002, 2013), career adaptability acts as psychosocial resources to cope with job demands, especially complex or unfavorable working conditions, or obstacles (Savickas, 2013). Although previous research has found some positive contextual factors to be predictors of career adaptability resources, little was known on how unfavorable working conditions and obstacles affect career adaptability, which yielded a critical theoretical void. As a set of obstacles in employees’ work environment, such as role conflict, role ambiguity (Kahn et al., 1964), situational constraints (Peters and O’Connor, 1980), work stressors are what we need to link to career adaptability, and address the above theoretical void. By empirically investigating the relationship between work stressors and career adaptability, the current research is the first to provide evidence on the proposed negative relationship between work stressors and career adaptability.

Third, by investigating the interaction effect of career adaptability and POS on CWB, we identified the compensatory effect between individuals’ adaptability resources and POS on affecting CWB. More importantly, we found that the compensatory role of POS was target-specific. That is, POS only moderated the relationship between career adaptability and CWB-O, but not the relationship between career adaptability and CWB-I. This result may advance the target similarity model (Lavelle et al., 2007) by exploring the target similarity in moderation analysis. Specifically, the main effect of POS on CWB-I (*r* = -0.24, *p* < 0.01) and CWB-O (*r* = -0.31, *p* < 0.01) were both significant, which showed no target similarity effect. However, when consider POS as a boundary condition in the relationship between career adaptability and CWB, the target similarity effect emerged.

### Practical Implications

The findings of the present research also provided some practical suggestions. First, organizations should consider the benefiting role of employees’ career adaptability. Employees with a high level of career adaptability will possess more resources to help them adjust to the environment better. Their concern over future careers, control over work-related actions and confidence to do the work well could help them regulate their behavior more efficiently and show lower levels of CWB both against the organization and coworkers. Since that career adaptability is less stable than personality traits (Savickas, 1997, 2013) and could be affected by both contextual factors and personal factors (Savickas and Porfeli, 2012), organizations may, on the one hand, set training programs that fostering employees’ career adaptability, and on the other hand prevent employees from the possible damage of career adaptabilities, such as reducing role ambiguity, role conflict, and other forms of work stressors around employees.

Second, as the detrimental effect CWB has on the individual and organizational outcomes, organizations should adopt multiple strategies to reduce the level of CWB. Besides implementing more efficient employee selection (Maclane and Walmsley, 2010), organizations could also directly, or indirect help employees exert control over their behavior. For instance, organizations may pay attention to the target-specific effect of organizational support on supplying the resources employees needed to exert better control over inappropriate behaviors. Because that organizational support is more effective for employees to reduce their CWB-O but not so effective to reduce their CWB-I, organizations may adopt additional ways of support, such as training employees to support coworkers. In this way, the coworker support may be especially useful for reducing the CWB-I.

### Limitations and Future Directions

We also consider some limitations and simultaneously suggest directions for future research. First, drawing upon career construction theory (Savickas, 2002, 2013), we argued that career adaptability mediates the relationship between work stressors and CWB above and beyond the mediation of social exchange, indicated by job satisfaction. Our research is an important supplement of the career construction theory. However, we acknowledge that, on the one hand, there are other indicators of social exchange, such as justice (Zellars et al., 2002; Kelloway et al., 2010). On the other hand, there are mediators from other theoretical perspectives, such as power (Popovich and Warren, 2010), attributions (Spector and Fox, 2010), and emotions (Spector and Fox, 2003). We recommend future studies incorporate other mediators from multiple perspectives in the relationship between work stressors and CWB. Doing so will provide a more comprehensive understanding of the mechanisms through which work stressors affects individuals’ extra-role behaviors.

Second, in the current study, we found the target-similarity effect of POS. Employees’ POS moderated the relationship between career adaptability and CWB-O but did not moderate the relationship between career adaptability and CWB-I. However, we did not measure moderators with the same foci of coworkers, such as perceived coworker support. Since that team members could influence individuals’ appraisal of stress (Carlijn et al., 2016), future studies could address this point and simultaneously examine the moderating effect of perceived organizational and coworker support on the relationship between career adaptability and both CWB-I and CWB-O. The ideal result may be that POS only moderates the career adaptability-CWB-O relationship and perceived coworker support only moderates the career adaptability-CWB-I relationship.

Third, besides supports from the organization (i.e., POS), future studies could also examine the role of other situational or personal moderators between stressors and CWB via career adaptability under the job demand-control-support model (Johnson and Hall, 1988). For instance, future research could take family or social support into consideration and examine the interaction effect of adaptability and family/social support on CWBs to see if family and social support have a similar effect with POS. Along with this path, future research may also include leadership into the model and investigate if support-providing leadership style such as supportive leadership (Paterson et al., 2014) or servant leadership (Liden et al., 2008) can be resources that compensate for employees’ personal resources. Besides, as career adaptability is a relatively unstable construct that providing resources (Savickas, 2002, 2013), future research could also examine if stable personality traits (e.g., grit; Ceschi et al., 2016) could aid or decrease personal control and interact with career adaptability in the relationship between stressors and CWB.

Fourth, although we used multi-wave design to reduce possible common-method variance (CMV), we admit that there may still be a concern of potential same-source bias. First, the independent variable (i.e., work stressors) and mediator (i.e., career adaptability) were measured at the same time (i.e., Time 1), while moderator (i.e., POS), and dependent variable (i.e., CWB) were measured at the same time (i.e., Time 2) due to practical reasons. Besides, all the measures in the current research were self-reported. Thus, we urge future research to adopt a longitudinal design and collected multi-source data (e.g., peer-rated CWB) to gain more solid causal inference.

Lastly, the data were collected in the Chinese e-commerce industry. As firms in the e-commerce industry must constantly adapt to the fast-changing environment in order to survive, employees within the industry are required to achieve job duties goals while also response quickly to dynamic market conditions (Wang et al., 2013), which may bring particularly high levels of stress to them. Moreover, the results that employees are performing CWBs was because of lacking self-regulation resources rather than the revenge motivation might be culturally specific, since that Chinese are to some extent more likely to inhibit aggression and less likely to take revenge than westerners (e.g., Maxwell et al., 2007). Therefore, future research could use data from other industries or cultures to validate the findings of the current study and explore cross-cultural implications further.

## Ethics Statement

This study was carried out in accordance with the recommendations of the Ethics guidelines of Institutional Review Board (IRB) of the School of Labor and Human Resources, Renmin University of China with written informed consent from all subjects. All subjects gave written informed consent in accordance with the Declaration of Helsinki. The protocol was approved by the Institutional Review Board (IRB) of the School of Labor and Human Resources, Renmin University of China.

## Author Contributions

All authors listed have made a substantial, direct and intellectual contribution to the work, and approved it for publication.

## Conflict of Interest Statement

The authors declare that the research was conducted in the absence of any commercial or financial relationships that could be construed as a potential conflict of interest. The reviewer LS declared a shared affiliation, with no collaboration, with the authors to the handling Editor at the time of review.
